# Ablation in Adult Congenital Heart Disease Using a Dual-Energy, Lattice-Tip, Large-Footprint, Map-and-Ablate Catheter

**DOI:** 10.1016/j.jaccas.2025.106130

**Published:** 2025-11-24

**Authors:** Salik ur Rehman Iqbal, Tobias Reichlin, Mathias Possner, Laurent Roten

**Affiliations:** Department of Cardiology, Inselspital, Bern University Hospital, University of Bern, Bern, Switzerland

**Keywords:** ablation, atrial fibrillation, atrial flutter, atrial tachycardia, congenital heart defect, electroanatomical mapping, electrophysiology, tetralogy of Fallot, transposition of the great arteries, ventricular tachycardia

## Abstract

**Background:**

Patients with adult congenital heart disease (ACHD) often develop atrial and ventricular arrhythmias. Ablation is challenging given complex anatomy and previous surgical repair. Pulsed-field ablation (PFA) offers improved efficacy and safety through myocardial selectivity. Novel catheters combining three-dimensional electroanatomical mapping with both PFA and radiofrequency ablation (RFA) streamline procedures in ACHD patients.

**Methods:**

We present a case series of 5 ACHD patients with symptomatic atrial and/or ventricular arrhythmias who underwent ablation using a dual-energy “map-and-ablate” lattice-tip catheter.

**Results:**

Using the lattice-tip catheter, pulmonary vein isolation, cavotricuspid isthmus ablation, and ablation of scar-related intra-atrial re-entry with combined RFA and PFA were performed in 2 patients and ablation of re-entrant ventricular arrhythmias in 3 patients. In 2 patients, both atrial and ventricular arrhythmias were ablated in the same procedure.

**Conclusions:**

Use of a large-area focal ablation catheter with mapping integration and dual-energy capabilities enables efficient and safe atrial and ventricular arrhythmia ablation in patients with ACHD.

## Graphical Abstract

**Figure:**
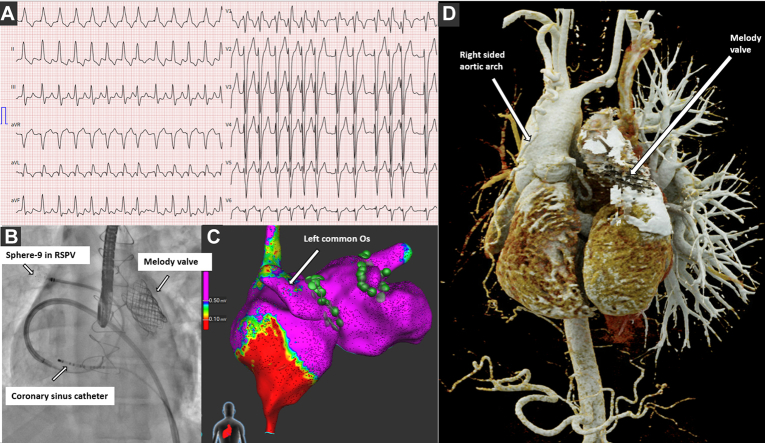

Patients with adult congenital heart disease (ACHD) commonly develop cardiac arrhythmias. Although catheter ablation is recommended as first-line therapy for recurrent arrhythmias, this approach remains challenging in many ACHD patients given complex anatomy and often extensive postsurgical scarring. Pulsed-field ablation (PFA) is a nonthermal, myocardium-selective ablation modality that spares adjacent noncardiac structures, causing less collateral damage.^1^ Few studies have reported on the safety and efficacy of PFA in ACHD patients, with those in publication primarily using the first-generation pentaspline PFA catheter.^2^^,^^3^

**Take-Home Messages**

• Ablation procedures in adult congenital heart disease can be simplified and made efficient with the use of a dual-energy lattice tip catheter with both mapping and ablating capabilities.
• PFA is emerging as a safe and effective ablation modality in congenital patients.

The Sphere-9 catheter (Affera, Medtronic) is a novel PFA catheter that integrates three-dimensional electroanatomical mapping (3D-EAM) capabilities and enables both radiofrequency ablation (RFA) and PFA. We report on our first experience using this catheter for ablation in ACHD patients with complex arrhythmias.

## Methods

In this case series, we included consecutive patients with complex ACHD who underwent ablation using the Sphere-9 catheter. All patients presented with symptomatic atrial fibrillation, atrial flutter (AFL), and or ventricular tachycardia (VT) (Table 1). In 3 patients, cardiac computed tomography was performed for anatomical assessment and procedural planning. All procedures were performed under general anesthesia. After obtaining ultrasound-guided femoral venous access, a decapolar catheter was positioned in the coronary sinus. In 2 patients, access to the pulmonary venous atrium was gained by transseptal puncture or transbaffle puncture using transesophageal echocardiography guidance. Unfractionated heparin was administered to maintain an activated clotting time of >350 seconds.

**Table 1:** Case Descriptions: Congenital Heart Disease and Previous Cardiac Interventions

Case	Age/Sex	Congenital Heart Disease	Previous Cardiac Interventions
1	46 y/M	Situs inversus totalis, AV and VA discordance, DORV, atresia of right PA, VSD, secundum-type ASD, right-sided aortic arch	1. Left-sided classic BTT shunt 2. Right-sided modified BTT shunt 3. VSD patch closure, tunneling of RV into aorta, suture closure of ASD, transsection and closure of proximal portion of PA, subpulmonic LV-PA conduit placement, patch enlargement of the right PA 4. Subpulmonic LV-PA conduit change 5. Implantation of Melody valve in subpulmonic LV-PA conduit
2	26 y/M	d-TGA, coronary anomaly (common ostium of the RCA with a retroaortic course and the LAD)	1. Atrial switch procedure (Mustard technique), PDA closure 2. Balloon dilation of baffle stenosis 3. Implantation of a cardiac monitor
3	38 y/M	d-TGA with pulmonary valve stenosis and VSD	1. Balloon atrial septostomy (Rashkind procedure) 2. Rastelli repair (VSD closure, LV-aorta connection, RV-PA reconstruction) 3. Implantation of an RV-PA conduit 4. Repeat surgical and transcatheter interventions on the RV-PA conduit 5. Ablation of IART, including CTI ablation 6. Reablation of CTI 7. Pulmonary veins isolation 8. CRT-D implantation for inducible ventricular tachycardia
4	33 y/M	DORV with Fallot physiology, right-sided aortic arch with abnormal branching of the vessels	1. Left-sided modified BTT shunt 2. Enlargement of the VSD with tunneling of the LV to the aorta, RVOT reconstruction with a conduit for pulmonary atresia, ligation of the atretic pulmonary trunk and partial PFO closure, epicardial PM implantation for complete AV block 3. Endovenous PM implantation owing to epicardial lead failure 4. Conduit replacement
5	59 y/M	Tetralogy of Fallot with right-sided aortic arch	1. VSD closure and resection of infundibular and valvular pulmonary stenosis 2. RVOT patch enlargement, repeat VSD closure, pulmonary valvuloplasty 3. Ablation of an incisional right atrial tachycardia and CTI ablation 4. Implantation of an RV/PA conduit, left and right pulmonary arterioplasty, closure of a residual VSD

ACHD = adult congenital heart disease; ASD = atrial septal defect; AV = atrioventricular; BTT = Blalock-Taussig-Thomas; CTI = cavotricuspid isthmus; DORV = double-outlet right ventricle; IART = intra-atrial re-entry tachycardia; LAD = left anterior descending artery; LV = left ventricle; M = male; PA = pulmonary artery; PDA = patent ductus arteriosus; PFO = patent foramen ovale; PM = pacemaker; RV = right ventricle; RVOT = right ventricular outflow tract; d-TGA = d-transposition of the great arteries; VA = ventriculoarterial; VSD = ventricular septal defect.

A 9 mm–diameter, compressible, lattice-tip catheter (Sphere-9) was used for high-densitiy 3D-EAM and dual-energy ablation with both RFA and PFA, as necessary (Table 2). For follow-up, all patients were instructed to undergo continuous 7-day Holter monitoring at 3, 6, and 12 months postablation. All patients provided written informed consent.

**Table 2:** Procedural Characteristics and Follow-Up

Case	Target Arrhythmia	Number of Lesions	Procedure Duration	Fluoroscopy Time	Radiation dose (cGy-cm^2^)	Ablation site(s)	Follow-Up
1^a^	AF, multiple IARTs	(1) 71 (PFA); 6 (RFA) (2) 35 (PFA); 75 (RFA)	(1) 275 min (2) 256 min	(1) 65 min (2) 30 min	(1) 14,865.98 (2) 3,687.2	(1) PVI (PFA); CTI (RFA) (2) Posteroseptal and inferior scar area in SVA; intercaval line; SVC isolation; roof line (PFA); PVI (PFA)	Acute hyperthyroidism with AF/AFL postablation; no further arrhythmias after antithyroid therapy and amiodarone
2	IART	42 (PFA); 17 (RFA)	146 min	8 min	57,462.08	Right PVI (RFA + PFA), Left PVI (PFA), CTI (RFA), septal scar region	9 mo via ICM; no arrhythmias
3	VT, IART, AF	34 (PFA); 47 (RFA)	216 min	11 min	291.94	RVOT scar, superior and lateral line from TV annulus to scar (RFA + PFA), AV node (RFA)	6 mo via CRT-D, no VTs
4	VT	42 (RFA), 7 (PFA)	168 min	13 min	359.41	Around RVOT scar and septal region (RFA + PFA), line from superior TV annulus to RVOT scar	Monomorphic VT postablation with cardioversion; upgrade to ICD; 5 mo via ICD without sustained VT
5	VT, IART	28 (RFA)	153 min	4 min	55.8	Line from VSD patch to PV and from RV incision to PV; CTI	No long-term follow-up available

AF = atrial fibrillation; AFL = atrial flutter; AV = atrioventricular; CRT-D = cardiac resynchronization therapy with defibrillator; CTI = cavotricuspid isthmus; IART = intra-atrial re-entry tachycardia; ICD = implantable cardioverter-defibrillator; ICM = implantable cardiac monitor; PFA = pulsed-field ablation; PV = pulmonary vein; PVI = pulmonary vein isolation; RFA = radiofrequency ablation; RV = right ventricular; RVOT = right ventricular outflow tract; SVA = systemic venous atrium; SVC = superior vena cava; TV = tricuspid valve; VSD = ventricular septal defect; VT = ventricular tachycardia.

^a^The patient in case 1 underwent 2 ablation procedures.

## Results

### Case 1

A 46-year-old man with situs inversus totalis and complex congenital heart disease presented with symptomatic atrial fibrillation requiring cardioversion (for details on prior interventions see Table 1). Medical therapy with amiodarone prevented recurrence for 11 months, until atrial fibrillation and AFL returned after discontinuation.

**Figure 1:**
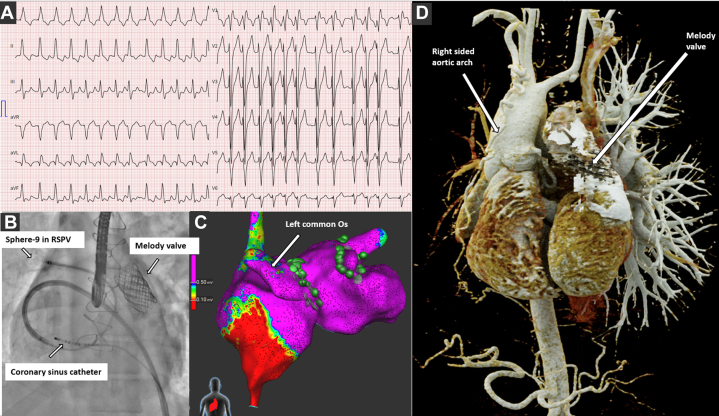
Findings on ECG and Initial Ablation Procedure in Case 1 (A) 12-lead ECG of clinical tachycardia. (B) Anteroposterior view showing the Sphere-9 catheter in the right superior pulmonary vein and the decapolar catheter in the coronary sinus. (C) Posteroanterior view showing pulmonary vein isolation using PFA (green dots). (D) Three-dimensional reconstruction of cardiac anatomy from computed tomography angiography. ECG = electrocardiogram; Os = ostium; PFA = pulsed-field ablation; RSPV = right superior pulmonary vein.

Using the Sphere-9 catheter, 3D-EAM of the systemic venous atrium (SVA) and, after a single transesophageal echocardiography–guided transseptal puncture, the pulmonary venous atrium (PVA) and pulmonary veins (PVs) was obtained, showing no scar. All four PVs were circumferentially ablated with PFA, achieving bidirectional block (Figure 1). In the SVA, cavotricuspid isthmus (CTI) ablation with RFA produced bidirectional block. Details of the procedure are in Table 2.

**Figure 2:**
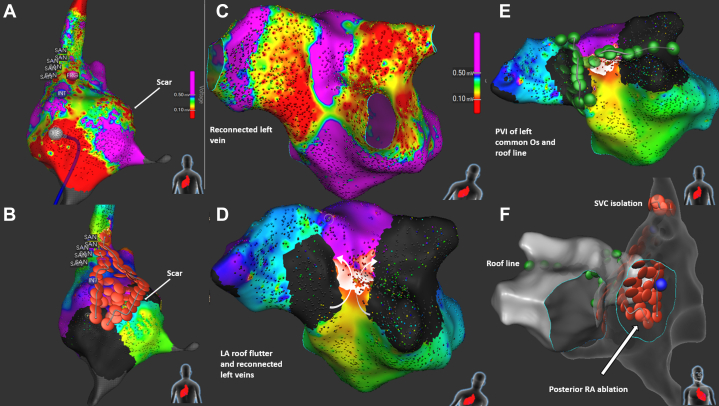
Second Ablation Procedure in Case 1 (A) Posteroanterior view of the systemic venous atrium showing low-voltage areas on the posterior septum. The region of the sinus atrial node (SAN) is tagged. (B) Radiofrequency ablation line with lesions connecting the superior vena cava to the scar, and covering the scarred septum. (C) Posterior view showing reconnected left vein and a narrow channel of healthy tissue on the posterior pulmonary venous atrium. (D) Activation map demonstrating roof-dependent flutter (arrows indicate direction of wavefront). (E) Posterior view showing PFA lesions (roof line and pulmonary vein isolation of left veins). (F) Anterior view showing combined lesion sets for systemic and pulmonary venous atria. LA = left atrial; Os = ostium; PFA = pulsed-field ablation; PVI = pulmonary vein isolation; RA = right atrial; SVC = superior vena cava.

Three weeks later, recurrent symptomatic AFL required repeated cardioversions. A second ablation using the Sphere-9 catheter confirmed persistent CTI block; 3D-EAM of the SVA showed extensive low-voltage areas posteroseptally and at the junction of the SVA and superior vena cava, with sinus node activation mapping confirming the mirror-image pattern of situs inversus (Figures 2A and 2B, Video 1). Multiple intra-atrial re-entry tachycardias (IARTs) were inducible and mapped to the SVA scar regions and a roof-dependent IART in the PVA (Figures 2B to 2D, Video 2). Left PV reconnection with a trigger from the left superior PV was also noted. RFA ablated the posteroseptal scar and created an intercaval line, followed by superior vena cava isolation after excluding phrenic nerve capture. The PVs were reisolated, and a posterior roof line was ablated using PFA (Figures 2E and 2F). No tachycardias were inducible at the end of the procedure.

Within 1 week postprocedure, the patient re-presented with atrial tachyarrhythmia requiring cardioversion. Work-up showed new-onset hyperthyroidism (thyroid function had been normal 2 months before the initial procedure); antithyroid therapy and beta-blockers were started, with no further recurrence during 3 months of follow-up.

### Case 2

**Figure 3:**
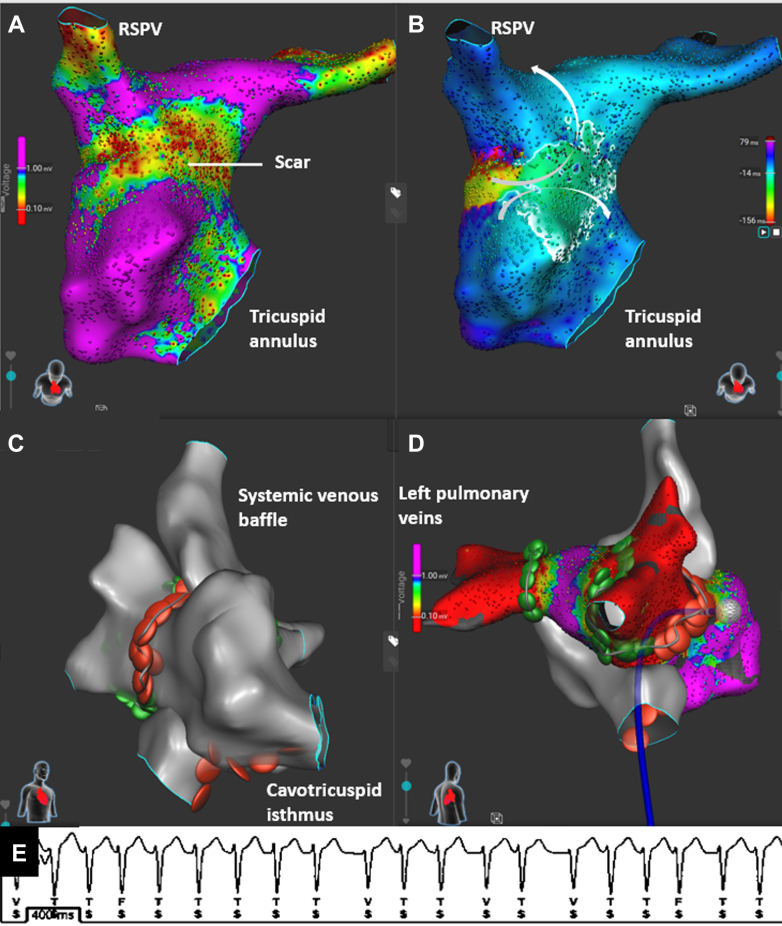
Ablation Procedure in Case 2 (A) Cranial view with voltage map of the pulmonary venous baffle. (B) Activation map of the tachycardia. Arrows (white) indicate the direction of the wavefront. (C) Right anterior oblique view showing CTI ablation (red dots) spanning both atria. (D) Pulmonary vein isolation around the right and left pulmonary veins with PFA (green dots) and RFA (red dots). (E) Rhythm strip from implantable cardiac monitor showing initial tachycardia. CTI = cavotricuspid isthmus; PFA = pulsed-field ablation; RFA = radiofrequency ablation; RSPV = right superior pulmonary vein.

A 26-year-old man with d-transposition of the great arteries (d-TGA) post–Mustard procedure reported recurrent palpitations. An implantable cardiac monitor documented IART, with ventricular rates up to 280 beats/min. An ablation procedure using the Sphere-9 catheter was performed, and the IART was induced. A 3D-EAM of both the SVA and, after a single transbaffle puncture, the PVA revealed a re-entry tachycardia around the right PVs, with a zone of slow conduction and septal scarring adjacent to the right PVs (Figures 3A and 3B, Video 3). After excluding phrenic nerve capture, both right PVs were isolated (PFA posteriorly, RFA anteriorly), terminating the tachycardia and restoring sinus rhythm. The left PVs were isolated using PFA, and CTI ablation was performed in both the PVA and SVA using RFA only, with confirmed bidirectional block (Figures 3C and 3D).^4^ No arrhythmias were detected during 9 months of implantable cardiac monitor follow-up.

### Case 3

**Figure 4:**
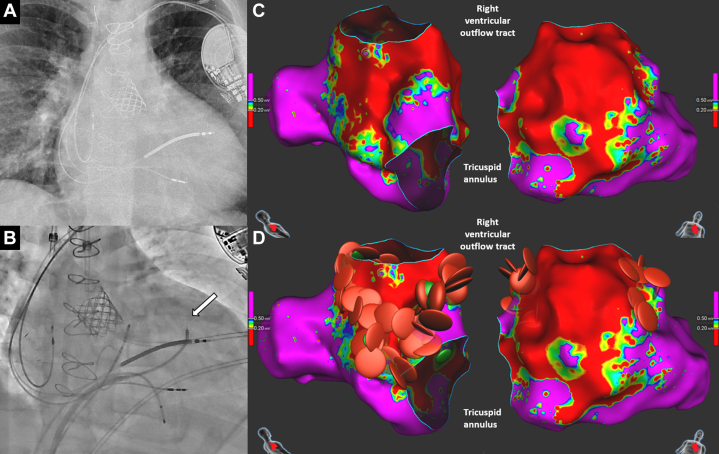
Findings on Chest X-Ray and Ablation Procedure in Case 3 (A) Anteroposterior chest x-ray showing cardiac resynchronization device and valve prosthesis in the right ventricular outflow tract. (B) Fluoroscopy image (right anterior oblique view) showing the position of the Sphere-9 catheter (arrow) against the high right ventricular septum. (C) Septal and lateral views showing scar areas. (D) Radiofrequency ablation lesions connecting the tricuspid annulus to the scar on the septal and lateral aspects.

A 38-year-old man with d-TGA and prior Rastelli repair, multiple interventions on the pulmonary valve and right ventricular outflow tract, implantation of cardiac resynchronization therapy with defibrillator for inducible VT with left bundle branch area pacing, and previous ablations for IARTs and atrial fibrillation (Figure 4A, Table 1), presented with symptomatic VT requiring an implantable cardioverter-defibrillator (ICD) shock. Recurrent episodes of IART and atrial fibrillation were also documented.

Clinical VT was induced with an inferior axis. Right ventricular voltage mapping with the Sphere-9 catheter revealed a large septal and anterosuperior scar (Figures 4C and 4D). Pace mapping within the scar reproduced the VT morphology with a long stimulus-to-QRS interval. Ablation lines from the superior and lateral tricuspid valve annulus to the scar were created using RFA and PFA. VT was no longer inducible.

Multiple IARTs were inducible, degenerating into atrial fibrillation. Atrioventricular node ablation was performed using the Sphere-9 catheter (RFA). No VTs occurred over 6 months.

### Case 4

**Figure 5:**
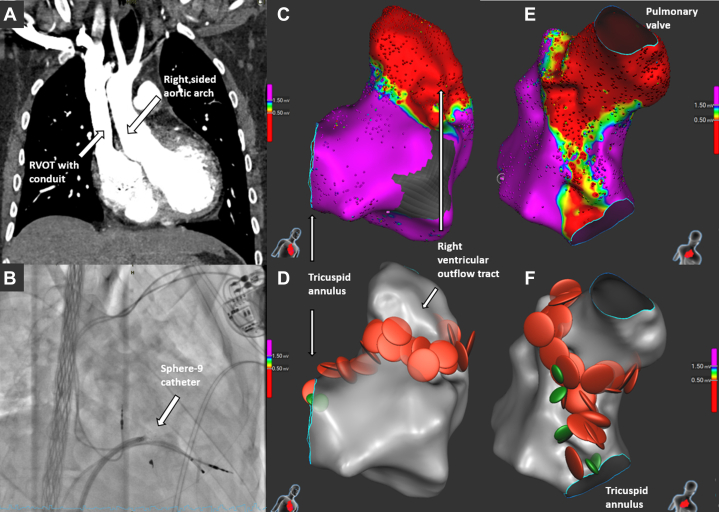
Findings on CT Angiography and Ablation Procedure in Case 4 (A) CT angiography anterior view showing right-sided aortic arch and RVOT conduit in a patient with double-outlet right ventricle and tetralogy physiology. (B) Right anterior oblique view showing the Sphere-9 catheter in the right ventricle. (C to F) RVOT scar (lateral and septal views) and RFA (red dots) and PFA (green dots) lesions encircling the scar boundary and connecting it to the tricuspid annulus. CT = computed tomography; PFA = pulsed-field ablation; RFA = radiofrequency ablation; RVOT = right ventricular outflow tract.

A 33-year-old man with repaired double-outlet right ventricle and prior pacemaker implantation for complete atrioventricular block had recurrent nonsustained monomorphic VT (up to 37 beats, 200-220 beats/min). Ventricular stimulation induced a hemodynamically unstable VT (inferior axis, left bundle branch block–like morphology, cycle length: 270 ms). The 3D mapping revealed a large anterior right ventricular outflow tract and high septal scar, the latter corresponding to the ventricular septal defect patch (Figures 5C to 5F). Pace mapping localized the VT origin to the high septal region. Circumferential right ventricular outflow tract scar ablation and a linear lesion to the superior tricuspid annulus were performed using RFA and PFA. VT was no longer inducible, but a monomorphic VT occurred hours later, requiring cardioversion. The pacemaker was upgraded to an ICD, and beta-blocker therapy was started. Over 5 months, no ICD interventions or sustained VT occurred.

### Case 5

**Figure 6:**
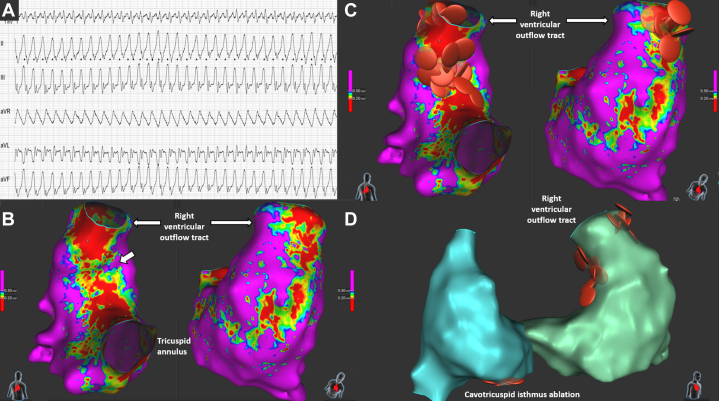
Findings on ECG and Ablation Procedure in Case 5 (A) ECG showing clinical ventricular tachycardia with inferior axis. (B) Septal and lateral views showing scarred areas and isthmus (small arrow). (C) Septal and lateral views showing RFA at both septal and lateral sites connecting the scar area to the right ventricular outflow tract. (D) Image showing combined atrial (cavotricuspid isthmus line) and ventricular RFA lesion. ECG = electrocardiogram; RFA = radiofrequency ablation.

A 59-year-old man with repaired tetralogy of Fallot and a history of ablation for incisional IART and CTI presented with wide-complex tachycardia. Electrophysiological study revealed inducible CTI-dependent flutter and 2 distinct, hemodynamically unstable ventricular arrhythmias (VT_1_: cycle length, 300 ms, superior axis; VT_2_: cycle length, 340 ms, right superior axis). CTI reablation was performed using the Sphere-9 catheter, followed by 3D-EAM and pace mapping of the right ventricle. Two classical isthmuses were identified as likely substrates for the 2 VTs: one from the ventricular septal defect patch to the pulmonary valve and another from an incisional right ventricular scar to the pulmonary valve (Figures 6B to 6D, Video 4). Both were ablated using RFA and PFA, after which VT was no longer inducible. No follow-up data were available at the time of manuscript submission.

## Discussion

This case series describes the use of a dual-energy, “map-and-ablate” lattice-tip catheter in complex ACHD patients. The lattice tip contains a magnetic sensor and 9 microelectrodes mounted on a compressible nitinol frame, which expands in the heart to form a 9 mm–diameter spherical array. This design enables rapid acquisition of a high-resolution electroanatomic map. It can also deliver radiofrequency energy in a temperature-controlled mode to create wide thermal lesions, and it can perform PFA using microsecond high-voltage pulses to generate nonthermal lesions. The large tip footprint and compliant design facilitates stable wall contact and efficient lesion formation, even in complex anatomies.

These features make the Sphere-9 catheter particularly well suited for the ablation of complex ACHD patients. Primarily, its “map-and-ablate” functionality eliminates the need for catheter exchanges. Understanding arrhythmia mechanisms in these patients typically requires detailed mapping of ongoing or induced arrhythmias followed by ablation, and multiple arrhythmias often require sequential mapping and ablation. Because access to certain chambers can be challenging in these patients, catheter exchange carries a risk of losing access. Catheter exchange has also been associated with prolonged procedural times and an increased risk of thromboembolic events.^5^ This was particularly advantageous in the second ablation procedure for case 1, where repeat mapping and ablation were required, and the procedure time would likely have been even longer with standard ablation catheters. Second, the complex and often-distorted anatomy may require ablation in close proximity to critical structures such as the esophagus or phrenic nerve, increasing the risk of adverse events (Table 3). The availability of PFA enables safe lesion delivery even in delicate regions of the heart, such as the posterior wall, as required in cases 1 and 2. Third, the arrhythmic substrate in these patients may be thick and resistant to conventional thermal ablation technologies. Radiofrequency ablation with the Sphere-9 catheter provides substantially greater power than standard RFA using conventional catheters. Furthermore, PFA can be applied in combination with RFA using the Sphere-9 catheter to enhance lesion durability, and linear ablations can be performed more efficiently than with previous catheter systems. The dual-energy capability also permits exclusive use of RFA in the vicinity of coronary arteries, as in case 2 to avoid spasm, for example during CTI ablation (Table 3).^6^ Other PFA catheters, such as the pentaspline PFA catheter, are less versatile and lack the dual-energy capability, making them less suitable for ablation in complex ACHD patients.^7^

**Table 3:** Possible Complications and Mitigation Strategies in RFA or PFA and How to Mitigate Using the Lattice-Tip Catheter

Possible Complications^a^	Ablation Energy and How to Mitigate
Esophageal injury	More common with RFA, switch to PFA during ablation of posterior wall
Phrenic nerve injury	More common with RFA, tag sites at or close to phrenic nerve and avoid ablating near them (if using RFA) or simply switch to PFA
Coronary spasms during CTI ablation	More common with PFA, switch to RFA during ablation. Alternatively, administer nitroglycerin as intracoronary (1 mg) or intravenous (1-2 mg) while using PFA^6^

CTI = cavotricuspid isthmus; PFA = pulsed-field ablation; RFA = radiofrequency ablation.

a No periprocedural or 30-day complications occurred in our patients using these mitigation strategies.

There is emerging evidence that PFA is effective and safe for the treatment of ventricular arrhythmias, as seen in cases 3 to 5.^8^^,^^9^ Preclinical studies have demonstrated deeper RFA lesions with the Sphere-9 catheter compared with a standard linear irrigated-tip catheter.^10^ In a study by Peichl et al,^11^ 81% of patients were free from ventricular arrhythmias at the 3-month follow-up. However, evidence on the use of PFA in the ventricle in complex ACHD patients remains insufficient. Krause et al^3^ reported a case of VT ablation in an Ebstein patient with an atrialized right ventricle using a pentaspline catheter. We present to our knowledge the first case series of VT ablation using the Sphere-9 catheter in ACHD patients. As with atrial arrhythmia, the catheter's “map-and-ablate” versatility and dual-energy capability are advantageous in patients with ventricular arrhythmia and are particularly useful when both atrial and ventricular arrhythmias must be treated, as in cases 3 and 5.

## Conclusions

The Sphere-9 catheter's combination of “map-and-ablate” functionality and dual-energy capability enables efficient, versatile, and safe ablation in complex ACHD patients. Its design reduces the need for catheter exchanges, facilitates lesion delivery near critical structures, and allows complementary use of RFA and PFA to optimize lesion durability. These attributes make it a valuable tool for managing challenging arrhythmic substrates in ACHD patients, particularly atrial arrhythmias. Though there is limited evidence for the role of PFA in ventricular arrhythmia, it seems a promising technology. Larger scale studies are required to evaluate its safety and efficacy.

## Funding Support and Author Disclosures

Dr Reichlin has received research grants from the Swiss National Science Foundation, the Swiss Heart Foundation, the sitem insel support fund, Biotronik, Boston Scientific, and Medtronic, for work outside the submitted study; speaker/consulting honoraria or travel support from Abbott/SJM, Biosense-Webster, Biotronik, Boston Scientific; and support for his institution's fellowship program from Abbott/SJM, Biosense-Webster, Biotronik, Boston-Scientific, and Medtronic. Dr Roten has received research grants from Medtronic, the Swiss National Foundation, the Swiss Heart Foundation, the Immanuel and Ilse Straub Foundation, and the Sitem Insel Support Fund, for work outside the submitted study; and speaker fees/honoraria from Biosense Webster, Boston Scientific, Abbott, and Medtronic. All other authors have reported that they have no relationships relevant to the contents of this paper to disclose.

## Appendix

Video 1 Intra-Atrial Re-Entry Tachycardia in the Systemic Venous Atrium in Case 1

Video 2 Roof-Dependent Intra-Atrial Re-Entry Tachycardia in the Pulmonary Venous Atrium in Case 1

Video 3 Intra-Atrial Re-Entry Tachycardia Around the Right Pulmonary Veins in Case 2

Video 4 Sinus Map Showing Areas of Late Activation in Case 5
